# B10 Promotes Polarization and Pro-Resolving Functions of Bone Marrow Derived Macrophages (BMDM) Through PD-1 Activation

**DOI:** 10.3390/cells14120860

**Published:** 2025-06-07

**Authors:** Takumi Memida, Guoqin Cao, Elaheh Dalir Abdolahinia, Sunniva Ruiz, Shengyuan Huang, Sahar Hassantash, Satoru Shindo, Motoki Okamoto, Shohei Yamashita, Shin Nakamura, Maiko Suzuki, Toshihisa Kawai, Xiaozhe Han

**Affiliations:** Department of Oral Science and Translational Research, College of Dental Medicine, Nova Southeastern University, 3301 South University Drive, Fort Lauderdale, FL 33314, USA; tmemida@nova.edu (T.M.); edalirab@nova.edu (E.D.A.); sruiz4@nova.edu (S.R.); dentisthsy@foxmail.com (S.H.); shassantash@outlook.com (S.H.); sshindo1@nova.edu (S.S.); mokamoto@nova.edu (M.O.); syamashi@nova.edu (S.Y.); shinnakamura06@okayama-u.ac.jp (S.N.); msuzuki@nova.edu (M.S.); tkawai@nova.edu (T.K.)

**Keywords:** regulatory B cell, B10 cells, macrophage, PD-1, SPMs

## Abstract

Regulatory B cells (B regs) are immune cells that help suppress excessive inflammatory responses by interacting with other immune components. Among them, B-10 cells are known for their strong immunoregulatory function. This study focused on how B-10 cells influence macrophage phenotype and function through the PD-1 signaling pathway. To investigate this, B-10 cells derived from mouse spleens were co-cultured with bone marrow-derived macrophages (BMDMs) from either wild-type (WT) or PD-1 knockout (PD-1 KO) mice, using both direct contact and Transwell setups. The findings indicated that direct co-culture with B-10 cells significantly promoted the polarization of macrophages towards the anti-inflammatory M2 type, characterized by increased expression of surface markers (F4/80^+^, CD206^+^, CD163^+^), higher levels of PD-1, and upregulation of M2-related genes (*IL-1ra*, *IL-10*, *Arg-1*, *IL-6*, and *CCL1*). These macrophages also exhibited enhanced phagocytic activity and greater secretion of specialized pro-resolving mediator (SPMs) like RvD2 and 15-epi LXA4. In contrast, these effects were reduced when B-10 cells were cultured indirectly or when PD-1 was absent. These findings suggest that B-10 cells promote anti-inflammatory macrophage activity primarily through PD-1 signaling, offering insights into potential therapeutic approaches for controlling inflammation.

## 1. Introduction

The regulatory B cells (B regs) have been studied for inhibiting excessive inflammation through immunoregulatory cytokines [1]. Notably, Interleukin-10 (IL-10) secreting B regs, termed B10 cells (B-10), exhibit pivotal roles in regulating immune responses through IL-10 production [2,3,4]. The previous article has shown that B-10 inhibits CD8^+^ T cell proliferation, and B-10 effector cell function can be enhanced through cognate interaction with CD4^+^ T cells via MHC classⅡand CD40 [5].

Macrophages are also known to be leading players in regulating the inflammatory state [6,7]. Generally, the pro-inflammatory macrophage (M1 phenotype) features pro-inflammatory mediators production, which hold out against microbial pathogens and activate the adaptive immune response [8]. In contrast, pro-resolving macrophage (M2 phenotype) polarizes to prevent the host from excessive inflammatory response and to facilitate the tissue repair process [6]. Macrophages also contribute critically to inflammation resolution through the phagocytic activity of neutrophils in apoptosis and the secretion of specialized pro-resolving mediators (SPMs) [9,10]. SPMs are synthesized from dietary polyunsaturated fatty acids (PUFAs) via lipoxygenase (LOX) and cyclooxygenase 1 and 2 (COX-1/2) [11] and contribute to the resolution of the inflammatory response. Emerging evidence has indicated that SPMs such as LipoxinA4 (LXA4) [12], ResolvinD1, D2 (RvD1 [13], RvD2 [14]), and ResolvinE1 (RvE1) [15] enhance the ability of the macrophage to clear apoptotic cells [16]. It is now recognized that enhancing pro-resolving function in the inflammatory microenvironment is crucial to tissue repair and regeneration [17].

We have already reported that B-10-rich CD1d^hi^ CD5^+^ B cells led to a significant decrease in inflammatory response and alveolar bone loss in a murine periodontitis model [18,19]. Moreover, we have described that the adaptation of B-10 cells treated with LPS and CpG in mice was infiltrated with more B-10 cells in the periodontium, promoting anti-inflammatory responses [20]. However, it remained unclear whether and how B-10 cells play a role in the inflammatory resolution through B-10–macrophage interaction. To elucidate the interaction between B-10 and macrophages, we explored the potential ligand–receptor connection between the two cell types. Interestingly, human CD19^+^CD24^+^CD38^+^ B cells, a subset of B regs, exhibit elevated programmed cell death-ligand 1 (PD-L1) expression [21]. On the other hand, programmed cell death 1 (PD-1), an immune checkpoint receptor, is expressed on regulatory T cells, B cells, dendritic cells, and macrophages and is well known for its inhibitory role in immune responses, including those related to cancer and autoimmune diseases [22,23]. Recently, it has been observed that PD-1-expressing macrophages display M2 function [24]. PD-L1/PD-1 axis represents a key ligand–receptor signaling pathway involved in negative immune regulation [25]. Thus, the crosstalk between PD-L1-expressing B-10 and PD-1-expressing macrophages may contribute to their pro-resolving functions via PD-L1/PD-1 engagement. Understanding how this pathway contributes to macrophage-mediated resolution of inflammation could provide valuable insight into the development of targeted therapies for chronic inflammatory and autoimmune diseases. In this context, B-10 cells, which produce IL-10 and express PD-L1, may be an attractive tool for cell-based immunotherapy. Thus, B-10 cell-based therapy could represent a novel and precise strategy for controlling pathological inflammation through macrophage-directed immune regulation.

In the present study, we investigated whether B-10 could potentiate macrophage pro-resolving function through PD-L1/PD-1 ligation. Our findings demonstrate that B-10 enhanced M2-type macrophage polarization, upregulated PD-1 expression, and pro-resolving functions such as phagocytic activity and specific SPM production. Such promotion of macrophage pro-resolving functions requires cell–cell contact, particularly via PD-1 activation.

## 2. Materials and Methods

### 2.1. Animals

Wild-type (WT) (C57BL/6) and PD-1 knockout (PD-1 KO) mice (B6.Cg-pdcd1tm1.1Shr/J) were obtained from Jackson Laboratory (Bar Harbor, ME, USA) and used in this study (6–8 weeks old). All mice were housed under specific pathogen-free conditions in cages equipped with air circulation systems. The animals were maintained at a temperature of 71 degrees Fahrenheit with 50 ± 20% relative humidity and exposed to a 14 h light and 10 h dark cycle. A standard rodent chow diet was provided. The use of laboratory animals in this study was approved by the IACUC of Nova Southeastern University.

### 2.2. Splenocyte Isolation and Culture

WT mice were sacrificed using a CO_2_ chamber, and their spleens were harvested in cold PBS. Splenocytes were mechanically dissociated using a glass syringe plunger and metal mesh. The cell suspension was centrifuged at 300× *g* for 5 min at room temperature (RT). Red blood cells were lysed using ACK Lysing Buffer (Gibco, Grand Island, NY, USA), with a 5 min incubation at RT. The lysate was filtered through a 40 μm cell strainer. After another centrifugation at 300× *g* for 5 min at room temperature (RT), the cell pellet was then resuspended in IMDM (Gibco, Grand Island, NY, USA) supplemented with 10% fetal bovine serum, 50 μM 2-mercaptoethanol (Gibco, Grand Island, NY, USA), and 1% antibiotic–antimycotic solution (Gibco, Grand Island, NY, USA). To produce IL-10 in B cells, splenocytes were treated with Porphyromonas gingivalis lipopolysaccharide (Pg. LPS) from ATCC33277 (10 μg/mL) (InvivoGen, San Diego, CA, USA), and CpG-TLR9 ligand (10 μM) (InvivoGen, San Diego, CA, USA). As previously described, the isolated cells were maintained with CpG and Pg.-LPS for 48 h [18,26]. After this period, the stimulated splenocytes were utilized for subsequent B-10 cell isolation.

### 2.3. Bone Marrow-Derived Macrophage Isolation and Culture

Mice were euthanized by CO_2_ inhalation, and the tibias and femurs were removed from WT and PD-1 KO mice. Bone marrow cells were collected by flushing the femoral and tibial bones and were cultured in DMEM (Gibco, Grand Island, NY, USA) containing 10% FBS, 1% Anti-Anti, 50 μM 2-ME (Medium A) with the addition of m-CSF (20 ng/mL, Biolegend, San Diego, CA, USA) for 2 days. After this period, the attached cells were collected as bone marrow-derived macrophages (BMDMs) and utilized for the subsequent co-culture procedure.

### 2.4. B-10 Culture and Isolation

B-10 cells were isolated using an established method [18]. Following the manufacturer’s protocol, B regs from wild-type (WT) mice were purified with the Regulatory B Cell Isolation Kit (Miltenyi Biotec, Cambridge, MA, USA). In brief, splenocytes were cultured with CpG-TLR9 and Pg. LPS for 48 h, supplemented with 50 ng/mL phorbol 12-myristate 13-acetate (PMA) and 500 ng/mL ionomycin during the final 5 h of stimulation. The splenic single-cell suspension was incubated at 4 °C for 10 min with a biotin–antibody cocktail targeting non-B cell markers, followed by a 15-min incubation at 4 °C with anti-biotin antibody-conjugated magnetic microbeads. Cells labeled with magnetic beads were removed using LD columns (Miltenyi Biotec, Bergisch Gladbach, Germany) within the QuadroMACS Separator (Miltenyi Biotec, Bergisch Gladbach, Germany). The unlabeled fraction, enriched for CD19^+^ cells with >99% purity, was collected.

For further purification of the B-10 cells, splenocytes depleted of non-B cell fraction were gently mixed into cold IMDM containing Regulatory B Cell Catch Reagent and harvested on ice for 5 min. The cell suspension (per 10^7^ cells) was then transferred to warm medium (37 °C) and incubated in a sealed tube at 37 °C for 45 min, with gentle agitation every 5 min to prevent cell settling. Post-incubation, cells were incubated with PE-conjugated Regulatory B Cell Detection Antibody on ice for 10 min, after which they were incubated for 15 min at 4 °C with anti-PE antibody-conjugated magnetic microbeads. Magnetically labeled cells were isolated using LS columns (Miltenyi Biotec, Bergisch Gladbach, Germany) under the QuadroMACS Separator. The collected cells, identified as B-10 cells, had a purity of >80% and were used for co-culture experiments.

### 2.5. Macrophage-B-10 Co-Culture

Adherent BMDMs were harvested by scraping and suspended in 10 mL of Medium A. The cell suspension was subjected to centrifugation at 300× *g* for 5 min, and the cells were resuspended. The cell number was determined using a hemacytometer. Naïve-B lymphocytes or B-10 were purified from mouse splenocytes as previously mentioned. BMDMs were co-cultured with either naïve-B or B-10 cells in 12 well plates, with or without Transwell (Greiner Bio-One, Kremsmünster, Austria), at a cell ratio of 1.0 × 10^5^ BMDMs to 5.0 × 10^5^ naïve-B or B-10 cells. Cultures were maintained at 37 °C in a 5% CO_2_ atmosphere for 48 h. Additionally, PD-1 knockout (KO) BMDMs were co-cultured with naïve-B cells or B-10 under the same conditions.

### 2.6. Flow Cytometry

Macrophage phenotypes and expression levels of PD-1 were assessed using flow cytometry. After co-culturing BMDM, cells were washed three times with PBS to remove the unattached cells (naïve-B lymphocytes or B-10). The experiment was performed on the isolated macrophages. BMDMs were collected and transferred to a U-bottom 96-well plate, then resuspended in PBS. The cells were stained with anti-mouse F4/80-APC, CD86-PE, CD206-PE, CD163-Brilliant Violet 421, and PD-1-PE antibodies (all from BioLegend, San Diego, CA, USA) and incubated at 4 °C for 30 min. Following staining, the cell suspension was centrifuged at 450× *g* for 5 min, and the supernatant was removed. After resuspension in PBS, the cell pellet was analyzed with a BD LSRFortessa X-20 flow cytometer (BD Biosciences, San Jose, CA, USA). Data analysis was performed using FlowJo software version 10.9.0 (FlowJo LLC, Ashland, OR, USA). Additionally, the representative gating strategy used in this study is shown in Supplementary Figure S2.

### 2.7. Western Blotting

Following the co-cultivation of BMDMs and either naïve-B lymphocytes or B-10, non-adherent cells were removed by washing three times with PBS. The experiment was conducted on the isolated macrophages. BMDMs were then lysed using lysis buffer (RIPA; Invitrogen, Carlsbad, CA, USA) in the presence of a protease inhibitor cocktail (Invitrogen, Carlsbad, CA, USA), followed by centrifugation at 15,000× *g* for 15 min. Protein concentrations in the collected supernatant were assessed via the BCA method. (Thermo Scientific, Waltham, MA, USA). Proteins from the lysates were separated by SDS-PAGE gels and transferred onto PVDF Transfer Stacks (Invitrogen, Carlsbad, CA, USA) using iblot 3. The membrane was blocked with 5% nonfat dry milk in TBS and probed overnight at 4 °C with primary antibodies against Anti-PD-1 IgG at a dilution of 1:250 (rabbit, PA5-20350 Invitrogen, Carlsbad, CA, USA) and anti-β-actin antibody at a dilution of 1:5000 (rabbit, 13E5, Danvers, MA, USA, Cell Signaling Technology, Danvers, MA, USA) followed by incubation with HRP-conjugated secondary antibodies. After a 5 min exposure to ECL substrate, images were acquired using an Azure C400 imaging system (Azure Biosystems, Dublin, CA, USA). Representative full gels are available in Supplementary Figure S1.

### 2.8. Real-Time RT-PCR

After 48 h of incubation of the BMDMs with naïve-B lymphocyte or B-10, non-adherent cells were removed by washing the BMDMs three times with PBS. The experiment was performed on isolated macrophages. Total RNA was then isolated from the BMDMs using Trizol reagent (Invitrogen, Carlsbad, CA, USA) and the PureLink RNA Mini Kit (Invitrogen, Carlsbad, CA, USA), according to the manufacturer’s protocol. Reverse transcription was performed using the cDNA Synthesis Kit (Verso) (Thermo Scientific, Waltham, MA, USA). Quantitative real-time PCR was carried out using PowerUp SYBR Green Master Mix (Applied Biosystems, Foster City, CA, USA) to assess the expression of genes including *Il10*, *Il1rn*, *Tgfβ*, *Vegfa*, *Il1β*, *Il6*, *Pdcd1*, *Arg1*, *Ccl1*, and *Alox15*. Gene expression fold changes were determined using the ΔΔCt method, with Gapdh as the housekeeping gene. Primer sequences used in this study are provided in Supplementary Table S1.

### 2.9. Phagocytosis Assay

The phagocytosis assay was performed only on macrophages after co-culture, using pHrodo Red S. aureus BioParticles Conjugate (Invitrogen, Carlsbad, CA, USA) according to the manufacturer’s instructions. In brief, pHrodo BioParticles Conjugate (25 μg/mL) was added to BMDM cultures after 48 h of co-culture, followed by a 30 min incubation at 37 °C. Phagocytosis was halted by placing the plates on ice. Unbound bioparticles were removed by washing the cells with PBS. Following detachment with StemPro Accutase (Gibco, Grand Island, NY, USA), cells were collected into round-bottom polystyrene tubes (Corning, NY, USA, Corning) and analyzed by flow cytometry using a BD LSRFortessa X-20 (San Jose, CA, USA, BD Biosciences). Imaging was performed with an EVOS M5000 system (Invitrogen, Carlsbad, CA, USA). Immunofluorescence images were acquired using a fluorescence microscope at 40× magnification. For each experimental condition, six fields per sample were randomly selected. Quantification of the fluorescence signal was analyzed using ImageJ software (version 1.54d, National Institutes of Health, Bethesda, MD, USA). A consistent threshold was applied across all images to exclude background fluorescence. The area of fluorescence-positive signals was measured and used as an index of fluorescence intensity. Approximately 200–300 cells were analyzed per condition. To ensure reproducibility, all measurements were conducted under identical image acquisition and processing settings.

### 2.10. LC-MS/MS-Based Lipidomics

After a 30 min incubation of BMDMs in high-glucose DMEM supplemented with HEPES and lacking phenol-red (Gibco, Grand Island, NY, USA), both culture supernatants and cells were transferred into cryovials and promptly frozen at −80 °C for later analysis.

Each 0.85 mL aliquot was mixed with 1 ng of internal standards (dissolved in 150 µL methanol), including 15(S)-HETE-d8, 14(15)-EpETrE-d11, Resolvin D2-d5, Leukotriene B4-d4, and Prostaglandin E1-d4, to enable accurate quantification and recovery assessment. The mixtures were thoroughly vortexed and subjected to the extraction of polyunsaturated fatty acid (PUFA) metabolites using C18 solid-phase columns, following established protocols [27,28]. The QTRAP7500 system was employed for mass spectrometric detection. In brief, the pre-spiked samples were loaded onto conditioned C18 cartridges, washed sequentially with 15% aqueous methanol and hexane, then dried under vacuum. Lipid mediators were eluted using 0.5 mL of methanol, evaporated under nitrogen, and the dried residue was reconstituted in 50 µL of a 1:1 solution of methanol and 25 mM ammonium acetate. Chromatographic separation was carried out on a Sciex UHPLC system equipped with a Luna C18 column (3 µm, 2.1 × 150 mm). A binary solvent system was used: Solvent A (methanol: water: acetonitrile = 10:85:5, *v*/*v*) and Solvent B (methanol: water: acetonitrile = 90:5:5, *v*/*v*), both containing 0.1% ammonium acetate. The gradient elution profile for Solvent B was programmed as follows: 50% (0–1 min), ramping to 80% (1–8 min), to 95% (8–15 min), and held at 95% (15–17 min), at a flow rate of 0.2 mL/min.

Eluates were directed into the QTRAP7500’s electrospray ionization source operating in negative ion mode with the following parameters: Curtain Gas at 40 psi, Ion Source Gases 1 and 2 at 40 and 70 psi, respectively, temperature at 600 °C, ion spray voltage at –2500 V, and low collision gas pressure. Lipid mediators were detected using a scheduled multiple reaction monitoring (MRM) strategy, targeting specific precursor–product ion pairs for each analyte. Collision energy (18–35 eV) and collision cell exit potentials (7–10 V) were optimized per transition.

For structural validation, Enhanced Product Ion (EPI) scans were recorded for each analyte. Chromatograms were processed using SciexOS 3.4 software. Quantification was performed based on the signal intensities of the internal standards, which served as normalization references for analyte recovery and relative abundance.

### 2.11. Statistical Analysis

Statistical analysis of the data was performed using Prism version 10 (GraphPad, La Jolla, CA, USA). An unpaired *t*-test was used to compare the differences between two independent groups. All data were presented as mean ± standard error (SEM). The criterion for statistical significance was *p* < 0.05.

## 3. Results

### 3.1. B-10 Cells Induce Macrophage Polarization Towards M2 Through Direct Cell–Cell Interaction

**Figure 1:**
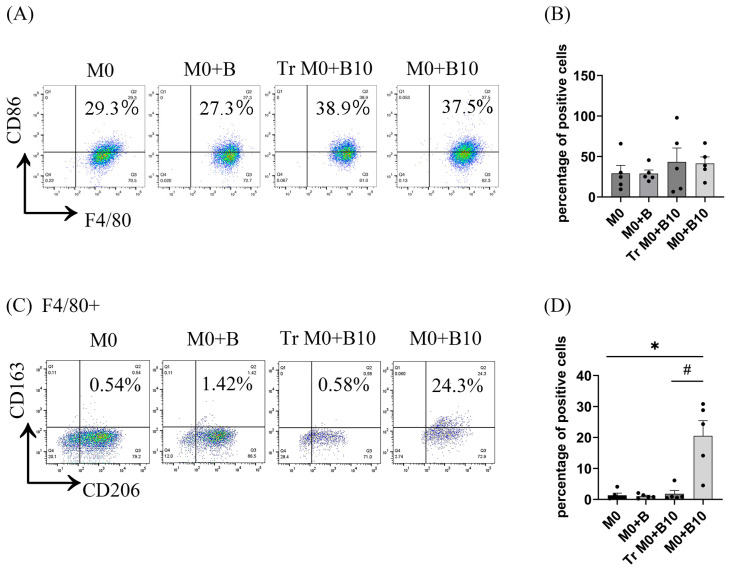
B-10 cells induce macrophage polarization towards M2 through direct cell–cell interaction. Bone marrow-derived macrophages (BMDMs) were harvested for 48 h with either naïve-B cells (B) or B-10 at a 1:5 ratio, either in direct contact or separated by a Transwell system (Tr). (**A**,**B**) The proportion of F4/80^+^ CD86^+^ cells was quantified by flow cytometry to identify the M1 phenotype. (**C**,**D**) The proportion of F4/80^+^ CD206^+^ CD163^+^ macrophages was evaluated by flow cytometry to identify the M2 phenotype. Experiments were independently replicated five times. Differences between groups were analyzed using an unpaired *t*-test. Data are presented as mean ± SEM, * *p* < 0.05 indicates significant differences compared to M0 alone (M0 + B, Tr M0 + B10, M0 + B10), while # *p* < 0.05 denotes a significant difference between direct and Transwell co-culture with B10 cells. Group descriptions: M0 (macrophages cultured alone), M0 + B (macrophages with naïve-B lymphocytes), Tr M0 + B10 (macrophages with B-10 in Transwell), M0 + B10 (macrophages directly co-cultured with B-10).

The ability of B-10 cells to influence macrophage polarization through direct cell–cell interaction was investigated. BMDMs were cultured with B-10 using a Transwell system to assess the role of physical contact. Transwell inserts were employed to prevent direct B-cell and macrophage interactions. Four experimental groups were established: M0 macrophages alone (M0), macrophages with naïve-B cells (M0 + B), macrophages with B-10 cells separated by Transwell (Tr M0 + B-10), and macrophages with B-10 cells in direct contact (M0 + B10). After 48 h of co-culture, the proportions of M1 macrophages (F4/80^+^/CD86^+^) and M2 macrophages (F4/80^+^/CD206^+^/CD163^+^) were evaluated by flow cytometry across all groups. The fraction of F4/80^+^ CD86^+^ cells showed no significant changes across all conditions (Figure 1A,B). In contrast, the proportion of F4/80^+^ CD206^+^ CD163^+^ cells in the M0 + B10 group (20.5 ± 4.93%) was markedly elevated in comparison with the M0 (1.22 ± 0.74%) (*p* < 0.05, Figure 1C,D). No appreciable changes in F4/80^+^ CD206^+^ CD163^+^ triple-positive cells were detected in the Tr M0 + B-10 or M0 + B groups relative to M0 alone. Additionally, the M0 + B-10 group (20.5 ± 4.93%) exhibited a significantly higher frequency of F4/80^+^ CD206^+^ CD163^+^ cells in comparison to the Tr M0 + B-10 group (1.72 ± 1.11%) (*p* < 0.05, Figure 1C,D). These results indicate that B-10 cells enhance M2 macrophage polarization through direct cell–cell contact, without influencing M1 macrophage differentiation.

### 3.2. B-10 Cells Upregulate PD-1 on Macrophages via Direct Cell–Cell Interaction

**Figure 2:**
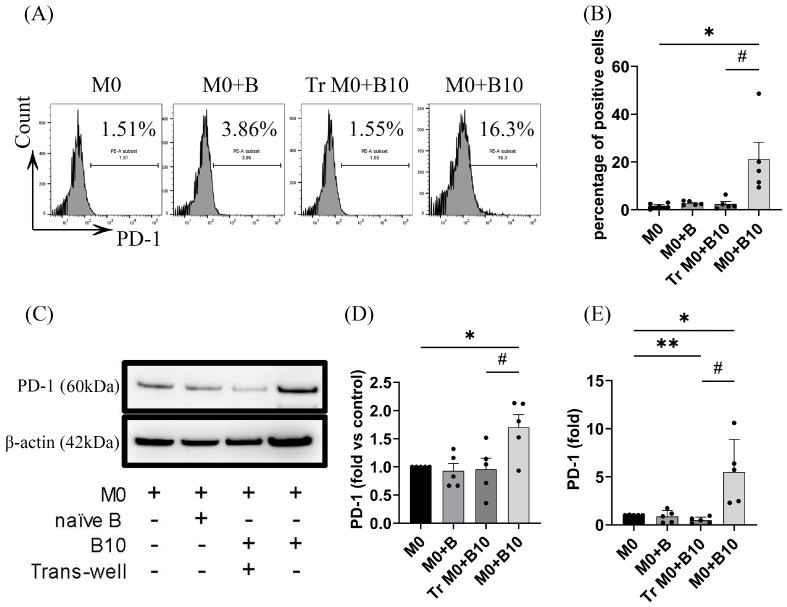
B-10 cells upregulate PD-1 on macrophages via direct cell–cell Interaction. Bone marrow-derived macrophages (BMDMs) were incubated with naïve-B lymphocytes (B) or B-10 (B-10) at a 1:5 ratio, either in direct contact or separated by a Transwell system (Tr), for 48 h. (**A**,**B**) Surface PD-1 expression on macrophages was assessed using PD-1-PE antibodies via flow cytometry. (**C**,**D**) PD-1 protein levels in BMDMs were quantified by immunoblotting. (**E**) *Pdcd1* gene expression in BMDMs was measured by real-time qPCR. Experiments were independently replicated five times. Group differences were evaluated using an unpaired *t*-test. Data are presented as mean value ± SEM, * *p* < 0.05, ** *p* < 0.01 (M0 + B, Tr M0 + B-10, and M0 + B-10 compared to M0-alone respectively), # *p* < 0.05 (M0 + B-10 compared to Tr M0 + B-10). Group descriptions: M0 (macrophages cultured alone), M0 + B (macrophages with naïve-B lymphocytes), Tr M0 + B10 (macrophages co-cultured with B-10 cells in Transwell), M0 + B10 (macrophages directly co-cultured with B-10).

We evaluated whether co-culturing BMDMs with B-10 cells could enhance PD-1 expression. PD-1 expression in BMDMs was assessed using quantitative PCR (qPCR), Western blotting, and flow cytometry. No significant changes in PD-1 protein or mRNA levels were observed in the M0 + B group compared to the M0 alone group. However, flow cytometry revealed a significant increase in PD-1 surface protein expression in the M0 + B10 group (19.2 ± 7.70) versus the M0 alone condition (*p* < 0.05, Figure 2A,B). Western blotting further confirmed elevated PD-1 protein levels in the M0 + B10 group (1.62 ± 0.20) relative to the M0-only condition (*p* < 0.05, Figure 2C,D). Similarly, qPCR revealed a marked increase in *Pdcd1* mRNA expression in the M0 + B-10 group (5.50 ± 1.52) compared to the M0 alone group (*p* < 0.05, Figure 2E). In contrast, the Transwell co-culture group (Tr M0 + B-10) did not show a significant increase in PD-1 protein levels when compared with the macrophage-only group (M0). Additionally, *Pdcd1* gene expression in the Tr M0 + B-10 condition was notably decreased (0.49 ± 0.14) relative to the M0-only condition (*p* < 0.05, Figure 2E). Importantly, both PD-1 gene and protein levels were considerably elevated in the M0 + B-10 group compared to the Transwell counterpart (*p* < 0.05, Figure 2A–E). Collectively, these results indicate that the upregulation of PD-1 in macrophages by B-10 cells requires direct intercellular contact.

### 3.3. B-10 Cells Enhance M2-Related Cytokines in Macrophages

**Figure 3:**
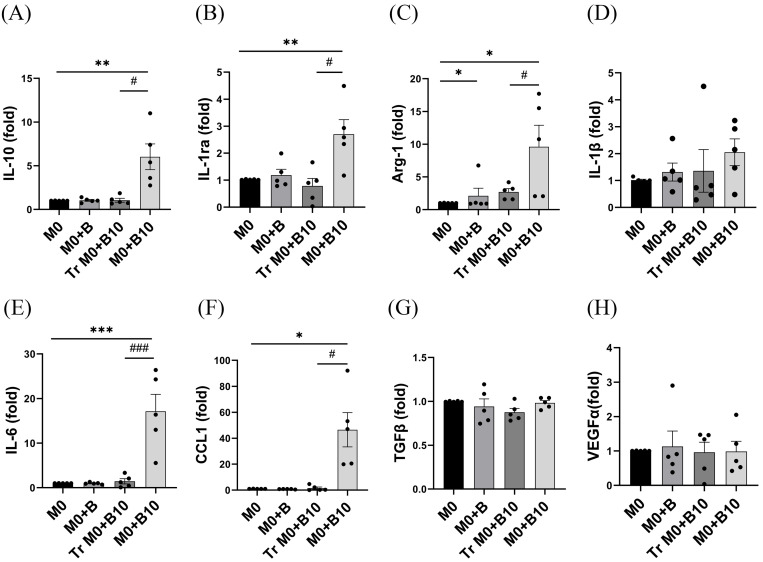
B-10 cells enhance M2-related cytokines in macrophages. A 48 h co-culture was conducted between BMDMs and either naïve-B lymphocytes or B-10 (1:5), using direct contact or Transwell-separated conditions. (**A**–**C**) Expression of M2a polarization marker (*Il10*, *Il1rn*, *Arg1*) was measured. (**D**–**F**) Expression of M2b-related genes (*Il1b*, *Il6*, *Ccl1*) was evaluated. (**G**) Expression of the M2c-related gene (*Tgfb*) was assessed. (**H**) Expression of the M2d-related gene (*Vegfa*) was quantified. All measurements were performed using real-time PCR. Experiments were performed independently on five separate occasions. Differences between groups were analyzed using an unpaired *t*-test. Data are presented as mean ± SEM, * *p* < 0.05, ** *p* < 0.01, *** *p* < 0.001 (M0 + B, Tr M0 + B10, and M0 + B10 in comparison with the M0-only respectively), # *p* < 0.05, ### *p* < 0.001 (direct vs. Transwell co-culture). Group descriptions: M0 (macrophages cultured alone), M0 + B (macrophages with naïve-B lymphocytes), Tr M0 + B10 (macrophages with B-10 cells in Transwell), M0 + B10 (macrophages directly co-cultured with B-10 cells).

Macrophages, classified into various subtypes, exhibit high plasticity and change their functional phenotypes in response to microenvironmental signals [29,30]. Notably, M2 macrophages are further subdivided into four subpopulations: M2a (IL-10^+^, IL-1ra^+^, and Arg-1^+^), M2b (IL-10^+^, CCL1^+^, IL-1β^+^, and IL-6^+^), M2c (IL-10^+^ and TGF-β^+^), and M2d (IL-10^+^ and VEGF-α^+^) [31]. As previously reported, B-10 cells promote M2 macrophage polarization through direct cell–cell contact. To explore which M2 subset-associated cytokines are upregulated in co-cultured macrophages, mRNA expression levels of *Il10* and various M2 subset markers were assessed, including *Il1rn* and *Arg1* as an M2a related marker, *Il1β*, *Il6*, *Ccl1* as a M2b related marker, *Tgfβ* as a M2c related marker, and *Vegfα* as a M2d related marker. No appreciable differences in cytokine expression were observed in the M0 + B group (M0 with naïve-B lymphocytes) in comparison with the M0-only condition. In the Tr M0 + B10 group (M0 with B-10 cells separated by Transwell), *Arg1* mRNA expression was significantly elevated (*p* < 0.05, Figure 3C). In contrast, the M0 + B10 group (M0 with B-10 cells in direct contact) exhibited significantly higher mRNA levels of *Il10*, *Il1rn*, *Arg1*, *Il6*, and *Ccl1* compared to the M0 alone group (*p* < 0.05, *p* < 0.01, or *p* < 0.001, Figure 3A–C,E,F). Moreover, when comparing the Tr M0 + B-10 and M0 + B-10 groups, M2a related genes (*Il10*, *Il1rn*, and *Arg1*) and M2b related genes (*Il6* and *Ccl1*) levels were markedly elevated in the M0 + B-10 (*p* < 0.05 or *p* < 0.001, Figure 3A–C,E,F). The present findings indicated that direct cell–cell contact was a crucial mechanism by which B-10 cells regulated the expression of M2a and M2b macrophage-related cytokines.

### 3.4. B-10 Cells Enhance Macrophage Phagocytosis Through Direct Cell–Cell Contact

**Figure 4:**
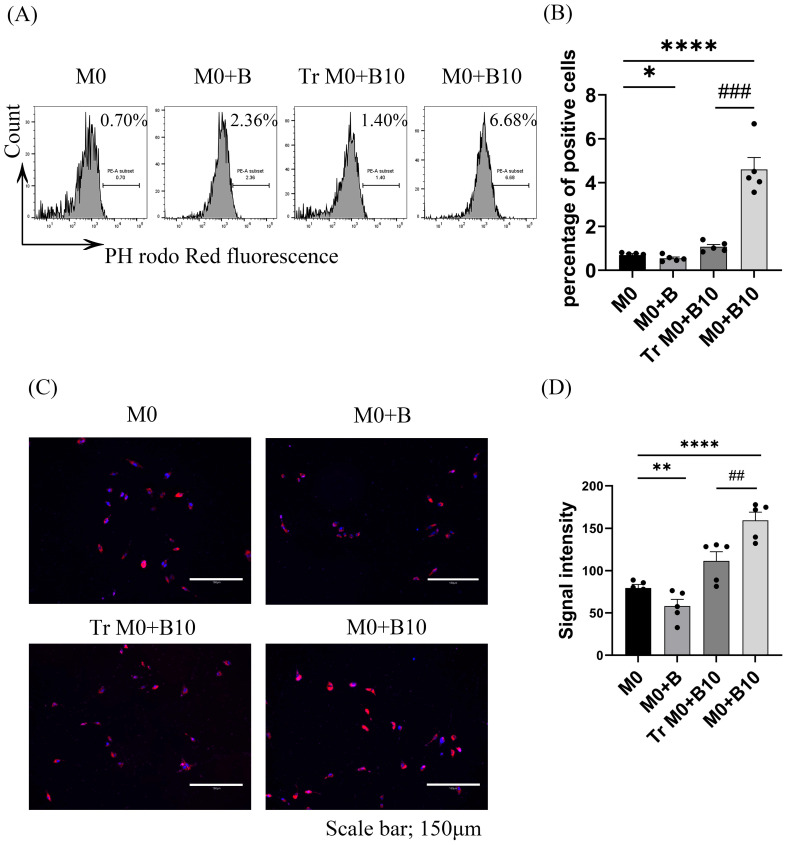
B-10 cells enhance macrophage phagocytosis through direct cell–cell contact. Bone marrow-derived macrophages (BMDM) were incubated with either naïve-B lymphocyte (B) or B-10 at a 1:5 ratio, either in direct contact or separated by a Transwell (Tr) system, for 48 h. Subsequently, 25 µg/mL of pH-sensitive fluorescent bioparticles was introduced into the culture medium, followed by a 30 min incubation at 37 °C. (**A**) Phagocytic cells were quantified using flow cytometry. (**B**) The proportion of pHrodo™ BioParticles™-positive macrophages was determined. (**C**) Representative images were captured using the EVOS M5000 imaging system. (**D**) Fluorescence intensity of the particles was measured using ImageJ software (version 1.54d). All experiments were independently replicated five times. Differences between groups were analyzed using an unpaired *t*-test. Data are presented as mean ± SEM, * *p* < 0.05, ** p < 0.01,**** *p* < 0.0001 (M0 + B, Tr M0 + B-10, and M0 + B-10 compared to M0-only respectively), ## *p* < 0.01, ### *p* < 0.001 (M0 + B-10 compared to Tr M0 + B-10). Group definitions: M0 (macrophages alone), M0 + B (M0 with naïve-B lymphocytes), Tr M0 + B10 (M0 with B-10 using Transwell), M0 + B10 (M0 with B-10 cells in direct contact).

Phagocytic activity, a critical pro-resolving aspect of macrophage activity, was evaluated to determine whether B-10 cells enhance this function. A phagocytosis assay was performed after co-culturing macrophages with B-10 cells. Cells were exposed to pHrodo™ Red *S. aureus* bioparticles for 30 min. Flow cytometry and immunofluorescent staining were subsequently used to assess phagocytic activity. Flow cytometry data revealed no change in the proportion of phagocytic cells in the M0 + B group. However, phagocytic activity was markedly increased in the Tr M0 + B-10 (1.20 ± 0.15), and the M0 + B-10 (4.35 ± 0.51) group exhibited significantly higher phagocytic activity compared to M0 alone (0.72 ± 0.06) (*p* < 0.05 or *p* < 0.0001, Figure 4A,B). Furthermore, when comparing M0 + B-10 and Tr M0 + B-10, the M0 + B-10 group exhibited significantly higher phagocytic activity (*p* < 0.0001). The immunofluorescent staining results showed that the pHrodo™ bioparticles-positive cells were increased dramatically in the Tr M0 + B-10 and M0 + B-10 groups compared to the M0 alone group (*p* < 0.01 and *p* < 0.0001, Figure 4C,D). Moreover, when comparing Tr M0 + B-10 and M0 + B-10 groups, more cells were observed in the M0 + B-10 (*p* < 0.01, Figure 4C,D). When comparing the Tr M0 + B-10 and M0 + B-10 groups, the M0 + B-10 group displayed a higher number of positive cells (*p* < 0.01, Figure 4C,D). These results, supported by immunofluorescence quantification, indicate that engulfing cells were more abundant in the direct B-10 culture condition. Collectively, these results suggest that B-10 cells enhance macrophage phagocytic function, primarily through direct interaction.

### 3.5. B-10 Cells Induce M2 Macrophage Polarization from M0 Through PD-L1/PD-1 Ligation

**Figure 5:**
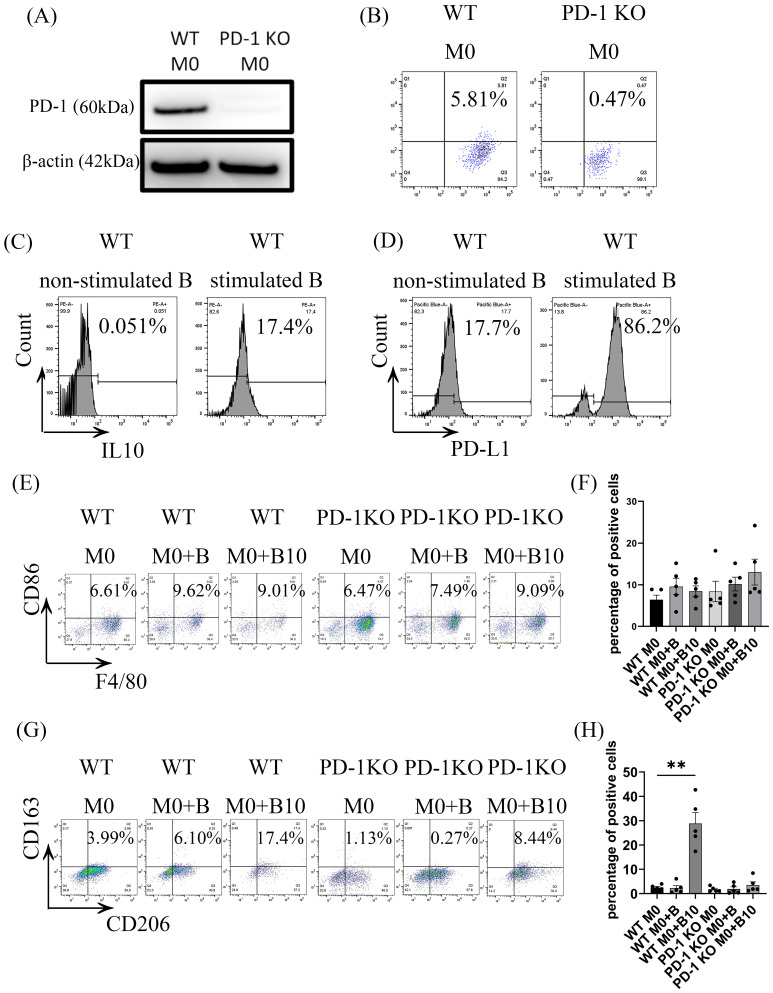
B-10 cells induce M2 macrophage polarization from M0 through PD-L1/PD-1 ligation. WT (C57BL/6) or PD-1 KO (B6.Cg-Pdcd1^tm1.1Shr^/J) BMDMs were cultured with B or B-10 in direct contact for 48 h. (**A**) PD-1 protein expression in WT and PD-1 KO BMDMs was quantified using Western blot analysis. (**B**) Surface PD-1 expression was assessed using flow cytometry with F4/80-APC and PD-1-PE antibodies (Q2 shows F4/80+ PD-1+ cells). (**C**) The proportion of CD19+ IL-10+ cells was measured using PE-A+ staining. (**D**) The proportion of CD19+ PD-L1+ cells was evaluated using Pacific Blue-A+ staining. (**E**,**F**) The proportion of F4/80+ CD86+ cells (M1 phenotype) was quantified using flow cytometry. (**G**,**H**) The percentage of F4/80+ CD206+ CD163+ cells (M2 phenotype) was quantified using flow cytometry. Experiments were independently replicated five times. Differences between groups were analyzed using an unpaired *t*-test. Data are presented as mean ± SEM, ** *p* < 0.01 (WT M0 + B, WT M0 + B10 compared to WT M0 alone, respectively). Group definitions: WT M0 (WT macrophages alone), WT M0 + B (WT M0 with naïve-B lymphocytes), WT M0 + B10 (WT M0 with B-10), PD-1 KO M0 (PD-1 KO macrophages alone), PD-1 KO M0 + B (PD-1 KO M0 with naïve-B cells), PD-1 KO M0 + B10 (PD-1 KO M0 with B-10 cells).

Macrophages polarized by B-10 cells exhibited an increase in PD-1 expression and a shift towards the M2 phenotype through direct cell–cell contact. However, the specific ligand-receptor interaction involved in this process remained unclear. We then investigated whether B-10 cells modulate the polarization of pro-resolving macrophages through PD-1 on macrophages and PD-L1 on B-10 cells. Firstly, we confirmed whether PD-1 was present in WT BMDMs and PD-1 KO BMDM. The results demonstrated that PD-1 was diminished in the PD-1 KO BMDMs (Figure 5A,B). The next experiments confirmed that PD-L1 expression on B-10 cells was enhanced by *Pg*.-LPS and CpG stimulation (Figure 5C,D). After 48 h of co-culture, we assessed M1 (F4/80+ CD86+) and M2 (F4/80+, CD206+, CD163+) phenotypic markers in the following groups using flow cytometry: WT M0, WT M0 + B, WT M0 + B10, PD-1 KO M0, PD-1 KO M0 + B, and PD-1 KO M0 + B10. The data revealed no consistent increase in the proportion of F4/80+ CD86+ cells in WT M0 + B or WT M0 + B10 groups versus the WT M0 condition, nor in PD-1 KO M0 + B or PD-1 KO M0 + B10 groups compared to PD-1 KO M0 alone (Figure 5E,F). While the population of F4/80^+^, CD206^+^, and CD163^+^ triple-positive cells showed no differences in WT M0 + B compared to WT M0 alone, a significant increase in F4/80^+^, CD206^+^, and CD163^+^ cells was observed only in the WT M0 + B10 cells group (25.63 ± 5.18%) in comparison with the WT M0-only condition (*p* < 0.01, Figure 5G,H). Nonetheless, no statistically significant differences were observed in the PD-1 KO M0 + B or PD-1 KO M0 + B10 group in comparison to the PD-1 KO M0 alone group (Figure 5G,H). This suggests that the PD-L1/PD-1 axis between B-10 cells and macrophages is essential to induce M2-type macrophage polarization.

### 3.6. PD-1/PD-L1 Ligation Regulates the Release of Cytokines Related to Various M2 Type Macrophages

**Figure 6:**
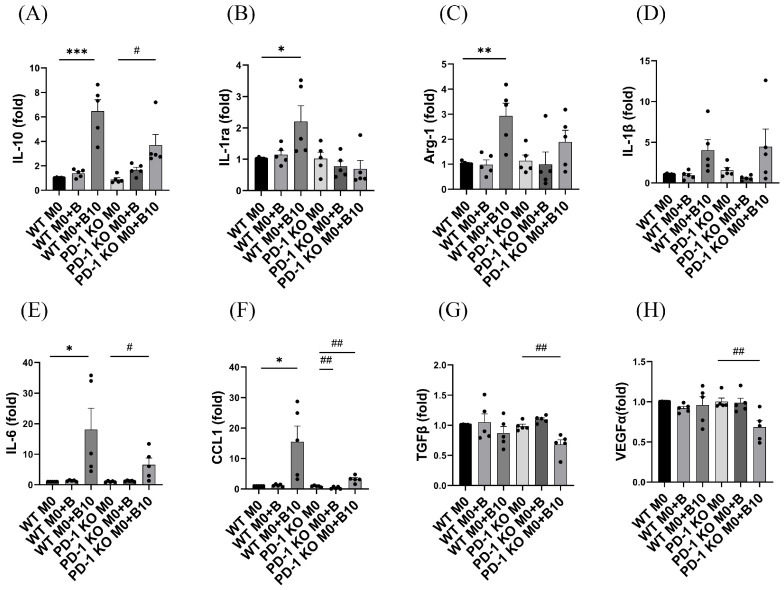
PD-1/PD-L1 ligation regulates the release of cytokines related to various M2-type macrophages. Bone marrow-derived macrophages (BMDMs) from wild-type C57BL/6 mice (WT M0) or PD-1 knockout mice (B6.Cg-Pdcd1tm1.1Shr/J; PD-1 KO M0) were co-cultured with naïve-B lymphocytes or B-10 for 48 h in direct contact. (**A**–**C**) Expression of M2a-related genes (*Il10*, *Il1rn*, *Arg1*) was quantified. (**D**–**F**) Expression of M2b-related genes (*Il1β*, *Il6*, *Ccl1*) was assessed. (**G**) Expression of the M2c related gene (*Tgfβ*) was evaluated. (**H**) Expression of the M2d-related gene (*Vegfα*) was measured. All measurements were performed using real-time qPCR. All experiments were conducted independently in five replicates. Differences between groups were analyzed using an unpaired *t*-test. Data are presented as mean ± SEM, * *p* < 0.05, ** *p* < 0.01, *** *p* < 0.001 (WT M0 + B, WT M0 + B10 compared to WT M0 alone, respectively), # *p* < 0.05, ## *p* < 0.01 (PD-1 KO M0 + B and PD-1 KO M0 + B10 compared to PD-1 KO M0 alone, respectively). Group definitions: WT M0 (WT macrophages alone), WT M0 + B (WT M0 with naïve-B lymphocytes), WT M0 + B10 (WT M0 with B-10 cells), PD-1 KO M0 (PD-1 KO macrophages alone), PD-1 KO M0 + B (PD-1 KO M0 with naïve-B cells), PD-1 KO M0 + B10 (PD-1 KO M0 with B-10 cells).

We next examined the role of PD-L1/PD-1 ligation in regulating M2-related cytokine expressions. Wild-type (WT) macrophages incubated with naïve-B cells showed no notable changes in cytokine mRNA levels in comparison to the WT M0 control. In contrast, WT BMDM cultured with B-10 displayed considerably elevated gene expression of M2a (*Arg1*, *IL1rn*, and *Il10*), M2b (*Ccl1* and *Il6*) relative to the WT M0 control (*p* < 0.05 or *p* < 0.001, Figure 6A–C,E,F). Conversely, PD-1 knockout (KO) macrophages cultured with naïve-B cells exhibited a significant reduction in CCL1 mRNA levels compared to the PD-1 KO M0 control (*p* < 0.01, Figure 3F). In PD-1 KO macrophages cultured with B-10 cells, mRNA levels of *Tgfβ* and *Vegfα* were markedly reduced (*p* < 0.01, Figure 6G,H), whereas *Il10*, *Il6*, and *Ccl1* mRNA levels were significantly elevated compared to the PD-1 KO M0 control (*p* < 0.05 or *p* < 0.01, Figure 6A,E,F). The present observations suggest the PD-L1/PD-1 interaction between B-10 cells and macrophages primarily enhances M2a-associated cytokine production, while M2b-associated cytokine expression appears unaffected by PD-L1/PD-1 signaling.

### 3.7. B-10 Cells Enhance Phagocytic Activity Through PD-L1/PD-1 Engagement

**Figure 7:**
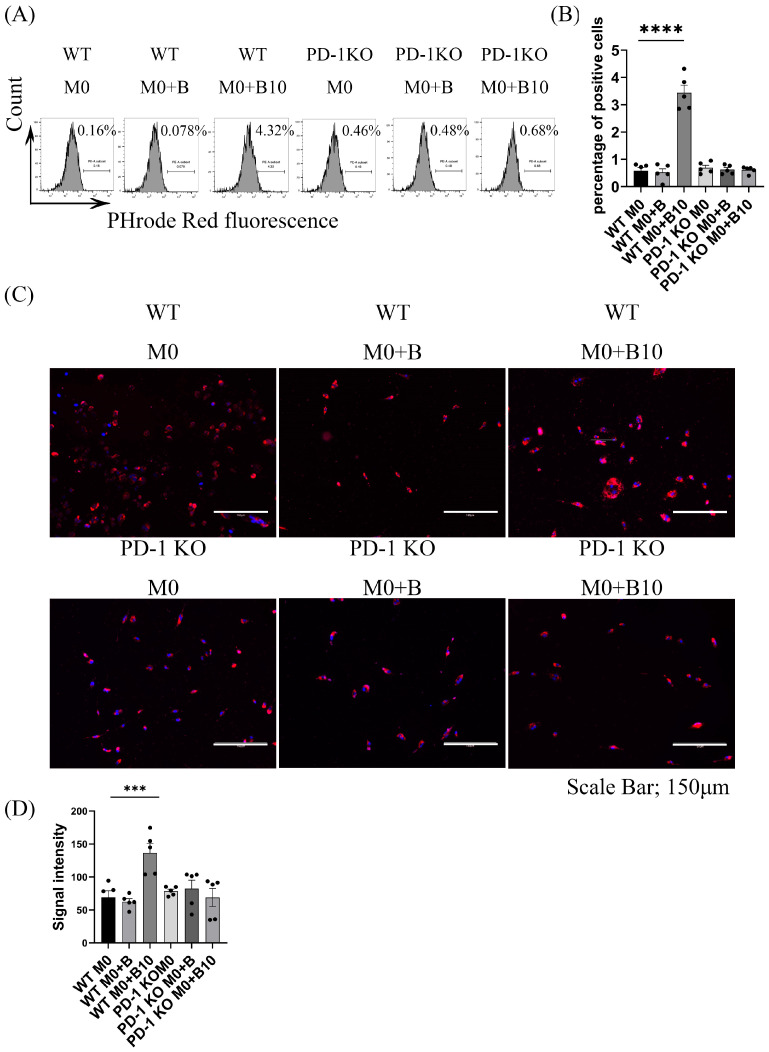
B-10 cells enhance phagocytic activity through PD-L1/PD-1 ligation. Bone marrow-derived macrophages (BMDMs) from wild-type C57BL/6 mice (WT M0) or PD-1 knockout mice (B6.Cg-Pdcd1^tm1.1Shr^/J; PD-1 KO M0) were co-cultured in direct contact with naïve-B lymphocytes or B-10 for 48 h. A concentration of 25 µg/mL pH-sensitive fluorescent bioparticles was applied to the culture and harvested for 30 min at 37 °C. (**A**) Phagocytic cells were evaluated using flow cytometric analysis. (**B**) The proportion of cells bearing pH-sensitive fluorescent bioparticles was determined. (**C**) Representative images were captured using the EVOS M5000 imaging system. (**D**) Fluorescence particle intensity was quantified using ImageJ software. All experiments were performed independently five times. Group differences were assessed using an unpaired *t*-test. Data are expressed as mean ± SEM, *** *p* < 0.001, **** *p* < 0.0001 (WT M0 + B, WT M0 + B10 compared to WT M0 alone, respectively). Group designations: WT M0 (WT macrophages alone), WT M0 + B (WT macrophages with naïve-B lymphocytes), WT M0 + B10 (WT macrophages with B-10), PD-1 KO M0 (PD-1 KO macrophages alone), PD-1 KO M0 + B (PD-1 KO macrophages with naïve-B cells), PD-1 KO M0 + B10 (PD-1 KO macrophages with B-10 cells).

We explored the contribution of PD-L1/PD-1 signaling to the phagocytosis induced by B-10 in macrophages. To eliminate PD-L1/PD-1 signaling, PD-1 KO macrophages were used in the co-culture experiments. A marked increase in the proportion of phagocytic macrophages was observed in WT macrophages co-cultured with B-10 cells relative to WT M0 alone (*p* < 0.0001) but not under co-culture conditions of WT BMDMs and naïve-B lymphocytes. In contrast, co-culture of PD-1 KO BMDM with naïve-B lymphocytes or B-10 reduced the percentage of phagocytic cells compared to the PD-1 KO M0 control group (Figure 7A,B). These observations were further supported by imaging and quantitative analysis (*p* < 0.001, Figure 7C,D). Collectively, these data indicate that B-10 facilitates macrophage phagocytic ability primarily via PD-L1/PD-1 ligation.

### 3.8. B-10 Cells Stimulate Specialized Pro-Resolving Mediator (SPM) Synthesis via PD-L1/PD-1 Interaction

**Figure 8:**
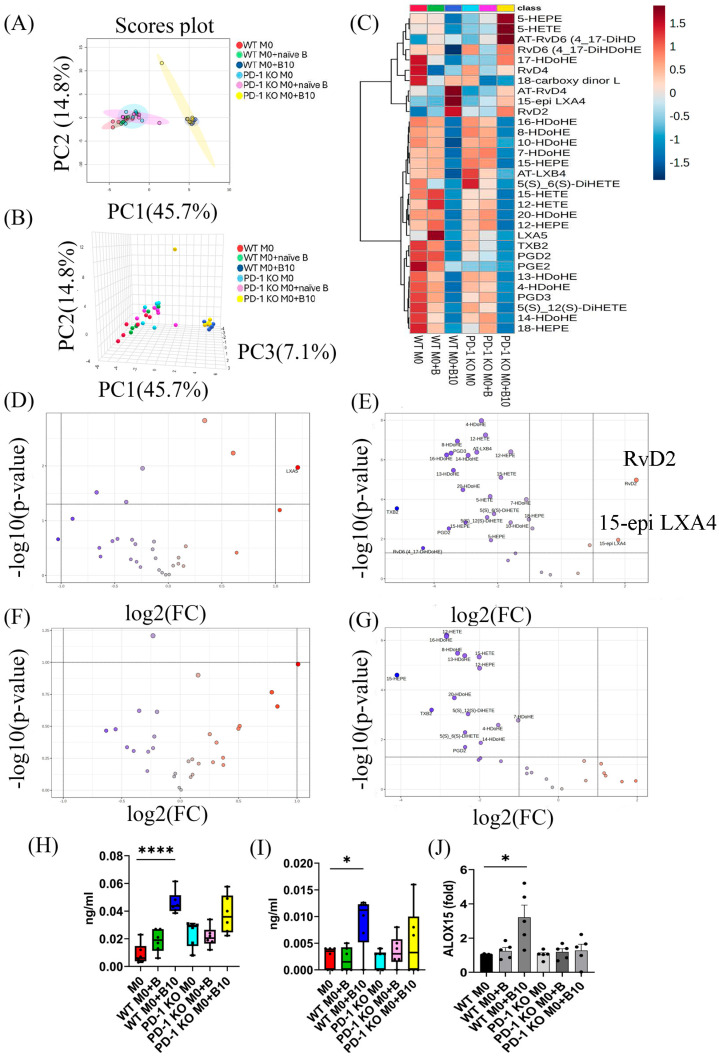
B-10 cells stimulate specialized pro-resolving mediator (SPM) synthesis via PD-L1/PD-1 interaction. Macrophages derived from bone marrow of wild-type C57BL/6 mice (WT M0) or PD-1 knockout mice (B6.Cg-Pdcd1tm1.1Shr/J; PD-1 KO M0) were incubated with naïve-B lymphocytes or B-10 for 48 h in direct contact. (**A**) Principal component analysis depicts group distinctions in 2D. (**B**) PCA illustrates group distinctions in 3D. Red dots represent WT M0 alone, green dots indicate WT M0 + B, blue dots signify WT M0 + B10, light blue dots denote PD-1 KO M0 alone, pink dots mark PD-1 KO M0 + B, and yellow dots indicate PD-1 KO M0 + B10. (**C**) Heatmap with hierarchical clustering visualizes SPM data. The color scale indicates the expression levels of SPMs, with darker colors representing higher expression. (**D**–**G**) Volcano plots combine fold change and *t*-tests to identify key SPM candidates. (**D**) WT M0 + B vs. WT M0, (**E**) WT M0 + B10 vs. WT M0, (**F**) PD-1 KO M0 + B vs. PD-1 KO M0, (**G**) PD-1 KO M0 + B10 vs. PD-1 KO M0. The *x*-axis represents log2 (fold change), and the *y*-axis represents -log10 (*p*-value). (**H**) Box plot quantifies RvD2 concentrations. (**I**) Box plot quantifies 15-epi LXA4 concentrations. (**J**) Expression of the *Alox15* gene was quantified using real-time qPCR. Independent replication was carried out five times for each experiment. Differences between groups were analyzed using an unpaired *t*-test. Data are presented as mean ± SEM, * *p* < 0.05, **** *p* < 0.0001 (WT M0 + B, WT M0 + B10 compared to WT M0 alone, respectively). Group definitions: WT M0 (WT macrophages alone), WT M0 + B (WT M0 with naïve-B lymphocytes), WT M0 + B10 (WT M0 with B-10 cells), PD-1 KO M0 (PD-1 KO macrophages alone), PD-1 KO M0 + B (PD-1 KO M0 with naïve-B cells), PD-1 KO M0 + B10 (PD-1 KO M0 with B-10 cells).

Specialized pro-resolving mediators (SPMs) are pivotal in mitigating inflammation. To assess whether B-10 cells drive SPM synthesis through PD-L1/PD-1 signaling, we analyzed lipidomics using liquid chromatography–tandem mass spectrometry (LC-MS/MS). Principal component analysis (PCA), depicted in 2D (Figure 8A) and 3D (Figure 8B) formats, revealed distinct SPM profiles between the two clusters. One cluster encompassed WT M0, WT M0 + B, PD-1 KO M0, and PD-1 KO M0 + B, while the other included WT M0 + B10 and PD-1 KO M0 + B10, with the B-10 co-culture conditions distinctly separated from others (Figure 8A,B). Heatmap analysis highlighted four SPMs—18-carboxy dinor LXB4, AT-RvD4, 15-epi LXA4, and RvD2—that were substantially higher in the presence of B-10 during co-culture compared to other groups (Figure 8C). Volcano plot analysis identified RvD2 (*p* < 0.05, Figure 8E,H) and 15-epi LXA4 (*p* < 0.05, Figure 8E,I) as dramatically upregulated in WT M0 + B-10 compared to WT M0-alone (Figure 8D–G). Notably, these elevated SPMs (RvD2 and 15-epi LXA4) were absent in PD-1 KO M0 + B and PD-1 KO M0 + B10 groups (Figure 8F,G). Furthermore, as the SPM pathway involves 15-lipoxygenase (15-LOX), we examined *Alox15* gene expression. No significant changes in *Alox15* mRNA levels were observed in WT M0 + B compared to WT M0 alone, but *Alox15* mRNA levels showed a significant increase in WT M0 + B-10 relative to WT M0 alone (*p* < 0.05, Figure 8J). In contrast, no differences were detected among PD-1 KO M0, PD-1 KO M0 + B, and PD-1 KO M0 + B10 groups (Figure 8J). These findings indicate that B-10 cells promote the synthesis of RvD2 and 15-epi LXA4, as well as *Alox15* gene expression, primarily through PD-L1/PD-1 signaling.

## 4. Discussion

The immunoregulatory role of B-10 cells is widely recognized to rely on the production of IL-10 [3,32]. Emerging research indicates that B regs regulate CD4+ T cells and T reg development through direct cell–cell interactions and cytokine-mediated pathways. Moreover, B regs have been shown to suppress CD8+ T cell expansion through specific cognate contacts [5]. These observations highlight the pivotal role of cell–cell interactions in mediating the anti-inflammatory properties of B regs. Nevertheless, the precise ligand-receptor mechanisms governing the regulatory effects of B-10 cells on macrophages remain unclear. In this study, we established that B-10 cells augment the inflammation-resolving capabilities of macrophages via PD-L1/PD-1 signaling.

To examine if a direct association between B-10 cells and macrophages is required for initiating a pro-resolving polarization state, we employed a Transwell system in co-culture experiments. Macrophages cultured in direct contact with B-10 cells exhibited increased expression of F4/80, CD206, and CD163, markers indicative of M2-type macrophages (Figure 1A–D). Furthermore, prior research has established that PD-1-expressing macrophages display M2-like surface characteristics [24]. Accordingly, we assessed PD-1 expression in macrophages. Our results revealed significantly elevated PD-1 levels in the M0 macrophage group that were co-cultured with B-10 cells (Figure 2A–E). These observations suggest that B-10 cells drive M2 macrophage polarization, marked by PD-1 expression, through direct cell–cell interactions.

In addition, in the PD-1 KO group, the percentage of M2 macrophages did not change even if co-cultured with B-10 (Figure 5A–D). Additionally, a prior study has shown that anti-PD-L1 treatment can encourage macrophages to adopt a more pro-inflammatory phenotype. Moreover, another article indicated that treating THP-1-derived macrophages with PD-L1 led to polarization toward M2-type macrophages [33]. Taken together, our results related to polarization toward M2-type macrophages align with these studies. Therefore, these findings suggest that PD-L1/PD-1 ligation between B-10 cells and macrophages plays a critical role in differentiating macrophages towards the M2 phenotype.

We then evaluated the effect of B-10 cells on M2-related macrophage cytokine expression. Macrophages are highly plastic and can change their functional phenotypes depending on microenvironmental signals. Notably, M2 macrophages are subdivided into four subpopulations: M2a, M2b, M2c, and M2d. Previous studies have shown that B-10 cells inhibit mRNA expression levels of *IL-1β* and *TNF-α*, pro-inflammatory related cytokines [26]. In this study, *IL-10* expression was significantly increased in macrophages co-cultured with B-10 cells directly, whereas this upregulation was markedly reduced when the cells were separated by a Transwell insert and in the PD-1 KO macrophage (Figure 3A and Figure 6A). Our observation supports that direct cellular contact, especially PD-L1/PD-1 engagement, plays a more crucial role than soluble factors in promoting IL-10 production in macrophages. Although B-10 cells are known to secrete IL-10 abundantly, the finding implies that IL-10 secretion by macrophages may be selectively induced through cognitive interactions. Furthermore, the present data also showed that while *Arg-1*, *IL-10*, and *IL-1ra*, anti-inflammatory related cytokines, exhibited a significant increase under direct co-culture conditions with B-10 cells (Figure 3A–C), unexpectedly, *IL-6*, a pro-inflammatory-associated cytokine, was also expressed by macrophages after co-culture with B-10 (Figure 3E). M2b macrophages, classified as regulatory macrophages, express CCL1 and IL-10 [31]. In addition to anti-inflammatory cytokines, M2b macrophages also secrete pro-inflammatory cytokines such as IL-1β, IL-6, and TNF-α [31,34]. M2b macrophages exhibit dual roles, contributing to both protective and pathogenic outcomes in disease contexts. Our findings suggest that *IL-6* expression is associated with M2b macrophages. Furthermore, in co-cultures with PD-1 knockout (KO) macrophages, levels of M2a-associated cytokines (*IL-1ra* and *Arg-1*) remained unchanged (Figure 6B,C), whereas M2b-related cytokines (*IL-6* and *CCL1*) were markedly elevated (Figure 6E,F). Conversely, M2c (*TGFβ*) and M2d (*VEGFα*) cytokines were reduced (Figure 6G,H). Currently, limited data exist regarding the link between M2b macrophage differentiation and the PD-L1/PD-1 signaling pathway. Recent studies have shown that when the PD-L1/PD-1 pathway was blocked, the population of M1 macrophages and pro-inflammatory cytokines, such as IL-1β and TNF-α, were increased [35]. However, these cytokines are expressed not only in M1 macrophages but also in M2b [31]. Furthermore, another research suggested that soluble PD-L1 secreted by trophoblast cells enhanced macrophage polarization towards the M2b phenotype, alleviating inflammation and facilitating tissue repair and tolerance [36]. Thus, PD-L1/PD-1 ligation appears necessary for the polarization toward M2a and M2b macrophages. However, our results showed that cell–cell contact could polarize macrophages to M2b, but this effect was not responsible for the PD-L1/PD-1 signaling. In this study, we demonstrated the different cytokine profile changes of M2 macrophages by using B cells. Since we utilized various co-culture systems with B cells to prevent cell–cell contact or PD-L1/PD-1 interactions, other cellular components may be involved in differentiating macrophages. Thus, these results suggested that macrophages co-cultured with B-10 cells exhibited upregulated mRNA levels of M2a, not responsible for M2b, M2c, and M2d specific genes, through PD-L1/PD-1 ligation.

Phagocytosis is recognized as a crucial mechanism in both innate and adaptive immune responses to foreign antigens, with macrophages playing a key role in promoting microbial clearance and wound debridement [37]. Recently, the function of lipid mediators (LMs) associated with the resolution of inflammation has been elucidated. These LMs, known as specialized pro-resolving mediators (SPMs), have been shown to enhance the phagocytic activity of macrophages [38]. In the current study, RvD2 and 15-epi LXA4 were identified as upregulated specific SPMs in macrophages co-cultured with B-10 cells (Figure 8C,E). Both RvD2 [39] and 15-epi LXA4 [40] are well known for their anti-inflammatory and pro-resolving effects. They also stimulate macrophage phagocytosis, thereby helping to modulate excessive immune responses. The engulfing function of macrophages co-cultured with B-10 cells was markedly increased compared to other groups without Transwell or with PD-L1/PD-1 signaling (Figure 4A–D and Figure 7A–D). These outcomes were in agreement with prior findings, which demonstrated that activation of the PD-1 pathway increased phagocytic activity [35]. In addition to direct quantification using the phagocytosis assay, the upregulation of CD163 observed in B-10-treated macrophages may further support enhanced phagocytic potential. CD163 is a well-characterized scavenger receptor involved in the uptake of apoptotic cells and hemoglobin–haptoglobin complexes, and its expression has been functionally linked to efficient clearance and inflammation resolution [41,42,43]. The increased levels of CD163 and the production of SPMs may indicate a coordinated transition towards pro-resolving macrophages that are capable of an efficient phagocytosis phenotype. These findings highlight a potential mechanism by which B-10 cells enhance macrophage function in the resolution phase of inflammation. Moreover, co-culturing B-10 with macrophages promoted the synthesis of RvD2 and 15-epi LXA4, which in turn activated the pro-resolving functions of macrophages. These SPMs are metabolized from DHA by 15-lipoxygenase (15-LOX). The murine enzyme 15-LOX, encoded by ALOX15, is implicated in the biosynthesis of several SPMs [44]. Previous studies have shown that the Th2 cytokines IL-4 and IL-13 are potent stimuli for the expression of 15-LOX in human monocytes [45]. As IL-4 strongly induces ALOX15 expression, 15-LOX is also recognized as an M2 macrophage marker [46]. In this study, *ALOX15* gene expression in macrophages was upregulated by co-culturing with B-10 (Figure 8J). The specific SPM production (RvD2 and 15-epi LXA4) was also increased (Figure 8H,I), and phagocytic activity in macrophages was enhanced (Figure 4A–D, Figure 7A–D). However, these results were diminished without cell–cell contact or PD-L1/PD-1 ligation. Little research has been conducted on PD-L1/PD-1 ligation and SPM production. The PD-L1/PD-1 axis is known as an immunosuppressive signal cascade, and that pathway leads to the polarization of M2-type macrophages [47]. SPMs are mainly synthesized by M2-type macrophages. Based on these results, it is suggested that B-10 cells enhanced the pro-resolving functions of macrophages, including phagocytic function and SPM secretion, through the PD-L1/PD-1 ligation.

As we described above, the reason why both M2a and M2b cytokines were upregulated was unclear. A previous study indicated that IL-10 was considered to enhance Fcγ receptor expression in monocytes and increase their responsiveness to immune complexes [48]. Immune complexes are known as one of the factors that polarize M2b-type macrophages. Therefore, the possible reason is that IL-10 produced by B-10 may reflect on the production of M2b-related cytokines. However, this study did not evaluate the effects of IL-10. Moreover, the previous article suggested that, in addition to IL-10 production and PD-L1 expression, other soluble factors or costimulatory molecules might be present in the regulatory B cells, including B-10 cells [49]. Moreover, several articles have reported the existence of pro-resolution macrophages (rM) [50]. The rM possesses a hybrid phenotype of M2 or M1. These macrophages express high ALOX15 and produce pro-resolving Lipoxin [51]. Such phenotypes are similar to macrophages co-cultured with B-10 directly in this study. In addition, although the precise downstream signaling was not examined, previous studies have demonstrated that STAT3 is a common effector activated by both IL-10 and PD-1 signaling. IL-10 has been reported to induce STAT3 activation, leading to M2 polarization of macrophages [52,53]. Moreover, PD-1^+^ M2-like tumor-associated macrophages have been shown to exhibit STAT3-mediated immunosuppressive programming, which can be reversed by PD-L1 blockade [54]. In addition to the STAT3 pathway, recent studies have suggested that PD-1 signaling may influence macrophage function through the PI3K/Akt axis. PD-1 has been shown to recruit SHP1/2 phosphatases, suppressing the PI3K/Akt pathway, thereby promoting a proinflammatory M1-like polarization state. In a murine model of lung ischemia–reperfusion injury, the inhibition of PD-1 was reported to restore PI3K/Akt activation in alveolar macrophages, resulting in reduced M1 polarization and inflammation [55]. Although this signaling mechanism was not directly examined in the present study, it is plausible that B-10-derived PD-L1 could suppress M1 polarization of macrophages via modulation of the PI3K/Akt pathway and STAT3 activation. This hypothesis warrants further investigation to better define the intracellular signaling networks that contribute to the pro-resolving effects induced by B-10 cells. Considering our results and these findings, it remains to be determined how other regulatory molecules, in addition to IL-10 and PD-L1, affect macrophage polarization and functions by B-10 cells.

In summary, this study demonstrated that PD-L1/PD-1 engagement increased the population of PD-1+ M2 macrophages and enhanced their phagocytic activity and production of specific SPMs (RvD2 and 15-epi LXA4) in primary bone marrow-derived macrophages from mice. We reported that the interaction between Raw 264.7 cells (a mouse macrophage cell line) and B-10 polarized pro-resolving macrophages enhances pro-resolving functions through cell–cell contact and IL-10 secretion [56]. In the current study, we utilized primary bone marrow-derived macrophages. We have observed different outcomes between these two cell types. However, we consistently demonstrated how B-10 cells influence bone marrow-derived macrophages to alleviate inflammatory responses, as mentioned above. In addition, PD-L1/PD-1 ligation is an essential cell component for B-10 cells to drive the pro-resolving function on macrophages. Based on these observations, it is the potential candidate that B-10 cells, which produce IL-10 and express PD-L1, could be used as a novel immunoregulatory therapy. In particular, the ability of B-10 cells to promote macrophage functions related to inflammation resolution may be useful for developing targeted therapies. Such cell-based approaches could be applied to control pathological inflammation in chronic inflammatory and autoimmune diseases, where immune regulation is often disrupted. Thus, a therapeutic strategy using B-10 cells may offer a precise and biologically relevant method to enhance macrophage-mediated immune resolution in the medical and dental field.

## 5. Conclusions

In conclusion, B-10 cells promote M2-like macrophages and enhance pro-resolving functions, such as phagocytic activity and specific SPM secretion, through PD-1 activation.

## Data Availability

The data presented in this study are available on request from the corresponding author.

## Supplementary Materials

The following supporting information can be downloaded at: https://www.mdpi.com/article/10.3390/cells14120860/s1. Figure S1.original gel images and microscope images (A, B) Original gel images corresponding to Figure 2C. (C, D) Original gel images corresponding to Figure 5A. (E) Original microscopy image corresponding to Figure 4C. (F) Original microscopy image corresponding to Figure 7C. Figure S2. Representative gating strategy for flow cytometric analysis of macrophage subsets and PD-1 expression. A sequential gating strategy was applied to identify macrophage populations and assess their polarization states. (A) Debris was excluded based on forward scatter area (FSC-A) and side scatter area (SSC-A). (B) Single cells were subsequently selected using an FSC-H versus FSC-A plot. (C) Macrophages were identified by gating F4/80^+^ cells. (D) Within the single-cell population, CD86 expression was analyzed in conjunction with F4/80 to define M1-like macrophages (F4/80^+^CD86^+^). (E) Within the F4/80^+^ population, M2-like macrophages were identified based on co-expression of CD206 and CD163. (F) PD-1 expression was also assessed within the F4/80^+^ gate to evaluate its expression on macrophages. Table S1. PCR primer sequences

## References

[1] Mauri C., Bosma A. (2012). Immune regulatory function of B cells. Annu. Rev. Immunol..

[2] Mizoguchi A., Mizoguchi E., Takedatsu H., Blumberg R.S., Bhan A.K. (2002). Chronic intestinal inflammatory condition generates IL-10-producing regulatory B cell subset characterized by CD1d upregulation. Immunity.

[3] Fillatreau S., Sweenie C.H., McGeachy M.J., Gray D., Anderton S.M. (2002). B cells regulate autoimmunity by provision of IL-10. Nat. Immunol..

[4] Carter N.A., Vasconcellos R., Rosser E.C., Tulone C., Muñoz-Suano A., Kamanaka M., Ehrenstein M.R., Flavell R.A., Mauri C. (2011). Mice lacking endogenous IL-10–producing regulatory B cells develop exacerbated disease and present with an increased frequency of Th1/Th17 but a decrease in regulatory T cells. J. Immunol..

[5] Yoshizaki A., Miyagaki T., DiLillo D.J., Matsushita T., Horikawa M., Kountikov E.I., Spolski R., Poe J.C., Leonard W.J., Tedder T.F. (2012). Regulatory B cells control T-cell autoimmunity through IL-21-dependent cognate interactions. Nature.

[6] Liu Y.-C., Zou X.-B., Chai Y.-F., Yao Y.-M. (2014). Macrophage polarization in inflammatory diseases. Int. J. Biol. Sci..

[7] Koh T.J., DiPietro L.A. (2011). Inflammation and wound healing: The role of the macrophage. Expert Rev. Mol. Med..

[8] Atri C., Guerfali F.Z., Laouini D. (2018). Role of human macrophage polarization in inflammation during infectious diseases. Int. J. Mol. Sci..

[9] Jordan P.M., Werz O. (2022). Specialized pro-resolving mediators: Biosynthesis and biological role in bacterial infections. FEBS J..

[10] Serhan C.N. (2014). Pro-resolving lipid mediators are leads for resolution physiology. Nature.

[11] Harwood J.L. (2023). Polyunsaturated fatty acids: Conversion to lipid mediators, roles in inflammatory diseases and dietary sources. Int. J. Mol. Sci..

[12] Prescott D., McKay D.M. (2011). Aspirin-triggered lipoxin enhances macrophage phagocytosis of bacteria while inhibiting inflammatory cytokine production. Am. J. Physiol.-Gastrointest. Liver Physiol..

[13] Krishnamoorthy S., Recchiuti A., Chiang N., Yacoubian S., Lee C.-H., Yang R., Petasis N.A., Serhan C.N. (2010). Resolvin D1 binds human phagocytes with evidence for proresolving receptors. Proc. Natl. Acad. Sci. USA.

[14] Chiang N., de la Rosa X., Libreros S., Serhan C.N. (2017). Novel resolvin D2 receptor axis in infectious inflammation. J. Immunol..

[15] Haas-Stapleton E.J., Lu Y., Hong S., Arita M., Favoreto S., Nigam S., Serhan C.N., Agabian N. (2007). Candida albicans modulates host defense by biosynthesizing the pro-resolving mediator resolvin E1. PLoS ONE.

[16] Decker C., Sadhu S., Fredman G. (2021). Pro-resolving ligands orchestrate phagocytosis. Front. Immunol..

[17] Vannella K.M., Wynn T.A. (2017). Mechanisms of organ injury and repair by macrophages. Annu. Rev. Physiol..

[18] Wang Y., Yu X., Lin J., Hu Y., Zhao Q., Kawai T., Taubman M.A., Han X. (2017). B10 cells alleviate periodontal bone loss in experimental periodontitis. Infect. Immun..

[19] Yu P., Hu Y., Liu Z., Kawai T., Taubman M.A., Li W., Han X. (2017). Local induction of B cell interleukin-10 competency alleviates inflammation and bone loss in ligature-induced experimental periodontitis in mice. Infect. Immun..

[20] Wang Y., Hu Y., Pan K., Li H., Shang S., Wang Y., Tang G., Han X. (2021). In-vivo imaging revealed antigen-directed gingival B10 infiltration in experimental periodontitis. Biochim. Et Biophys. Acta (BBA)-Mol. Basis Dis..

[21] Hasan M.M., Thompson-Snipes L., Klintmalm G., Demetris A.J., O’Leary J., Oh S., Joo H. (2019). CD24hiCD38hi and CD24hiCD27+ human regulatory B cells display common and distinct functional characteristics. J. Immunol..

[22] Liang S.C., Latchman Y.E., Buhlmann J.E., Tomczak M.F., Horwitz B.H., Freeman G.J., Sharpe A.H. (2003). Regulation of PD-1, PD-L1, and PD-L2 expression during normal and autoimmune responses. Eur. J. Immunol..

[23] Azuma T., Yao S., Zhu G., Flies A.S., Flies S.J., Chen L. (2008). B7-H1 is a ubiquitous antiapoptotic receptor on cancer cells. Blood J. Am. Soc. Hematol..

[24] Gordon S.R., Maute R.L., Dulken B.W., Hutter G., George B.M., McCracken M.N., Gupta R., Tsai J.M., Sinha R., Corey D. (2017). PD-1 expression by tumour-associated macrophages inhibits phagocytosis and tumour immunity. Nature.

[25] Iwai Y., Hamanishi J., Chamoto K., Honjo T. (2017). Cancer immunotherapies targeting the PD-1 signaling pathway. J. Biomed. Sci..

[26] Cao G., Xu Q., Huang S., Dai D., Wang J., Li W., Zhao Y., Lin J., Han X. (2024). B10 cells regulate macrophage polarization to alleviate inflammation and bone loss in periodontitis. J. Periodontol..

[27] Maddipati K.R., Zhou S.-L. (2011). Stability and analysis of eicosanoids and docosanoids in tissue culture media. Prostaglandins Other Lipid Mediat..

[28] Maddipati K.R., Romero R., Chaiworapongsa T., Zhou S.-L., Xu Z., Tarca A.L., Kusanovic J.P., Munoz H., Honn K.V. (2014). Eicosanomic profiling reveals dominance of the epoxygenase pathway in human amniotic fluid at term in spontaneous labor. FASEB J..

[29] Mosser D.M., Edwards J.P. (2008). Exploring the full spectrum of macrophage activation. Nat. Rev. Immunol..

[30] Mantovani A., Sozzani S., Locati M., Allavena P., Sica A. (2002). Macrophage polarization: Tumor-associated macrophages as a paradigm for polarized M2 mononuclear phagocytes. Trends Immunol..

[31] Wang L.-X., Zhang S.-X., Wu H.-J., Rong X.-L., Guo J. (2019). M2b macrophage polarization and its roles in diseases. J. Leukoc. Biol..

[32] Iwata Y., Matsushita T., Horikawa M., DiLillo D.J., Yanaba K., Venturi G.M., Szabolcs P.M., Bernstein S.H., Magro C.M., Williams A.D. (2011). Characterization of a rare IL-10–competent B-cell subset in humans that parallels mouse regulatory B10 cells. Blood J. Am. Soc. Hematol..

[33] Wei Y., Liang M., Xiong L., Su N., Gao X., Jiang Z. (2021). PD-L1 induces macrophage polarization toward the M2 phenotype via Erk/Akt/mTOR. Exp. Cell Res..

[34] Ito I., Asai A., Suzuki S., Kobayashi M., Suzuki F. (2017). M2b macrophage polarization accompanied with reduction of long noncoding RNA GAS5. Biochem. Biophys. Res. Commun..

[35] Zhang Y., Ma L., Hu X., Ji J., Mor G., Liao A. (2019). The role of the PD-1/PD-L1 axis in macrophage differentiation and function during pregnancy. Hum. Reprod..

[36] Zhang Y.-H., Aldo P., You Y., Ding J., Kaislasuo J., Petersen J.F., Lokkegaard E., Peng G., Paidas M.J., Simpson S. (2020). Trophoblast-secreted soluble-PD-L1 modulates macrophage polarization and function. J. Leucoc. Biol..

[37] Greenlee-Wacker M.C. (2016). Clearance of apoptotic neutrophils and resolution of inflammation. Immunol. Rev..

[38] Fredman G., Khan S. (2023). Specialized pro-resolving mediators enhance the clearance of dead cells. Immunol. Rev..

[39] Spite M., Norling L.V., Summers L., Yang R., Cooper D., Petasis N.A., Flower R.J., Perretti M., Serhan C.N. (2009). Resolvin D2 is a potent regulator of leukocytes and controls microbial sepsis. Nature.

[40] Mitchell S., Thomas G., Harvey K., Cottell D., Reville K., Berlasconi G., Petasis N.A., Erwig L., Rees A.J., Savill J. (2002). Lipoxins, Aspirin-Triggered Epi-Lipoxins, Lipoxin Stable Analogues, and the Resolution of Inflammation: Stimulation of Macrophage Phagocytosis of Apoptotic Neutrophils: In Vivo. J. Am. Soc. Nephrol..

[41] Kneidl J., Löffler B., Erat M.C., Kalinka J., Peters G., Roth J., Barczyk K. (2012). Soluble CD163 promotes recognition, phagocytosis and killing of Staphylococcus aureus via binding of specific fibronectin peptides. Cell. Microbiol..

[42] Jiao K., Zhang J., Zhang M., Wei Y., Wu Y., Qiu Z.Y., He J., Cao Y., Hu J., Zhu H. (2013). The identification of CD163 expressing phagocytic chondrocytes in joint cartilage and its novel scavenger role in cartilage degradation. PLoS ONE.

[43] Schulz D., Severin Y., Zanotelli V.R.T., Bodenmiller B. (2019). In-depth characterization of monocyte-derived macrophages using a mass cytometry-based phagocytosis assay. Sci. Rep..

[44] Sezin T., Ferreirós N., Jennrich M., Ochirbold K., Seutter M., Attah C., Mousavi S., Zillikens D., Geisslinger G., Sadik C.D. (2020). 12/15-Lipoxygenase choreographs the resolution of IgG-mediated skin inflammation. J. Autoimmun..

[45] Ivanov I., Kuhn H., Heydeck D. (2015). Structural and functional biology of arachidonic acid 15-lipoxygenase-1 (ALOX15). Gene.

[46] Radmark O. (2022). Formation of eicosanoids and other oxylipins in human macrophages. Biochem. Pharmacol..

[47] Li W., Wu F., Zhao S., Shi P., Wang S., Cui D. (2022). Correlation between PD-1/PD-L1 expression and polarization in tumor-associated macrophages: A key player in tumor immunotherapy. Cytokine Growth Factor Rev..

[48] van Roon J., Wijngaarden S., Lafeber F.P., Damen C., van de Winkel J., Bijlsma J.W. (2003). Interleukin 10 treatment of patients with rheumatoid arthritis enhances Fc gamma receptor expression on monocytes and responsiveness to immune complex stimulation. J. Rheumatol..

[49] Blair P.A., Noreña L.Y., Flores-Borja F., Rawlings D.J., Isenberg D.A., Ehrenstein M.R., Mauri C. (2010). CD19+ CD24hiCD38hi B cells exhibit regulatory capacity in healthy individuals but are functionally impaired in systemic lupus erythematosus patients. immunity.

[50] Bystrom J., Evans I., Newson J., Stables M., Toor I., Van Rooijen N., Crawford M., Colville-Nash P., Farrow S., Gilroy D.W. (2008). Resolution-phase macrophages possess a unique inflammatory phenotype that is controlled by cAMP. Blood J. Am. Soc. Hematol..

[51] Stables M.J., Shah S., Camon E.B., Lovering R.C., Newson J., Bystrom J., Farrow S., Gilroy D.W. (2011). Transcriptomic analyses of murine resolution-phase macrophages. Blood J. Am. Soc. Hematol..

[52] Williams L., Bradley L., Smith A., Foxwell B. (2004). Signal transducer and activator of transcription 3 is the dominant mediator of the anti-inflammatory effects of IL-10 in human macrophages. J. Immunol..

[53] Sarma U., Maiti M., Nair A., Bhadange S., Bansode Y., Srivastava A., Saha B., Mukherjee D. (2021). Regulation of STAT3 signaling in IFNγ and IL10 pathways and in their cross-talk. Cytokine.

[54] Han Z., Wu X., Qin H., Yuan Y.-C., Schmolze D., Su C., Zain J., Moyal L., Hodak E., Sanchez J.F. (2023). Reprogramming of PD-1+ M2-like tumor-associated macrophages with anti–PD-L1 and lenalidomide in cutaneous T cell lymphoma. JCI Insight.

[55] He X., Xiao J., Li Z., Ye M., Lin J., Liu Z., Liang Y., Dai H., Jing R., Lin F. (2023). Inhibition of PD-1 alters the SHP1/2-PI3K/Akt axis to decrease M1 polarization of alveolar macrophages in lung ischemia–reperfusion injury. Inflammation.

[56] Memida T., Abdolahinia E.D., Cao G., Ruiz S., Huang S., Shindo S., Nakamura S., Lin J., Kawai T., Han X. (2025). B10 cells promote pro-resolving macrophage function through direct cell-cell contact and IL-10 secretion in Raw 264.7 cells. Int. Immunol..

