# Supplementary material for: B10 Promotes Polarization and Pro-Resolving Functions of Bone Marrow Derived Macrophages (BMDM) Through PD-1 Activation
Source: Cells. 2025 Jun 7;14(12):860. doi: 10.3390/cells14120860 (PMC12191351; doi:10.3390/cells14120860)
Supplement: Supplementary file 1 [file cells-14-00860-s001.zip › cells-3653153-supplementary.pdf]

Supplementary figure

Fig.S1

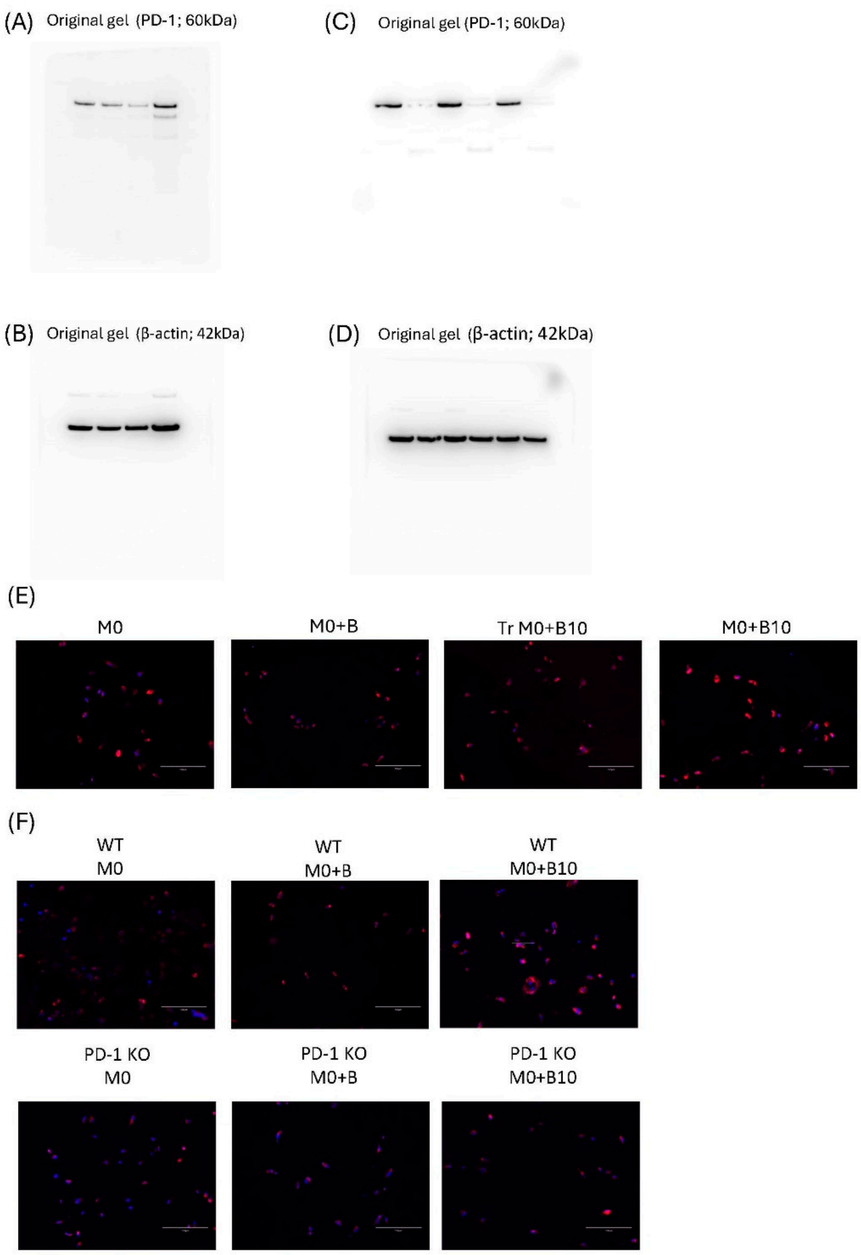

Fig.S1 original gel images and microscope images

(A, B) Original gel images corresponding to Fig. 2C. (C, D) Original gel images corresponding to Fig. 5A. (E) Original microscopy image corresponding to Fig. 4C. (F) Original microscopy image corresponding to Fig. 7C.

Fig.S2

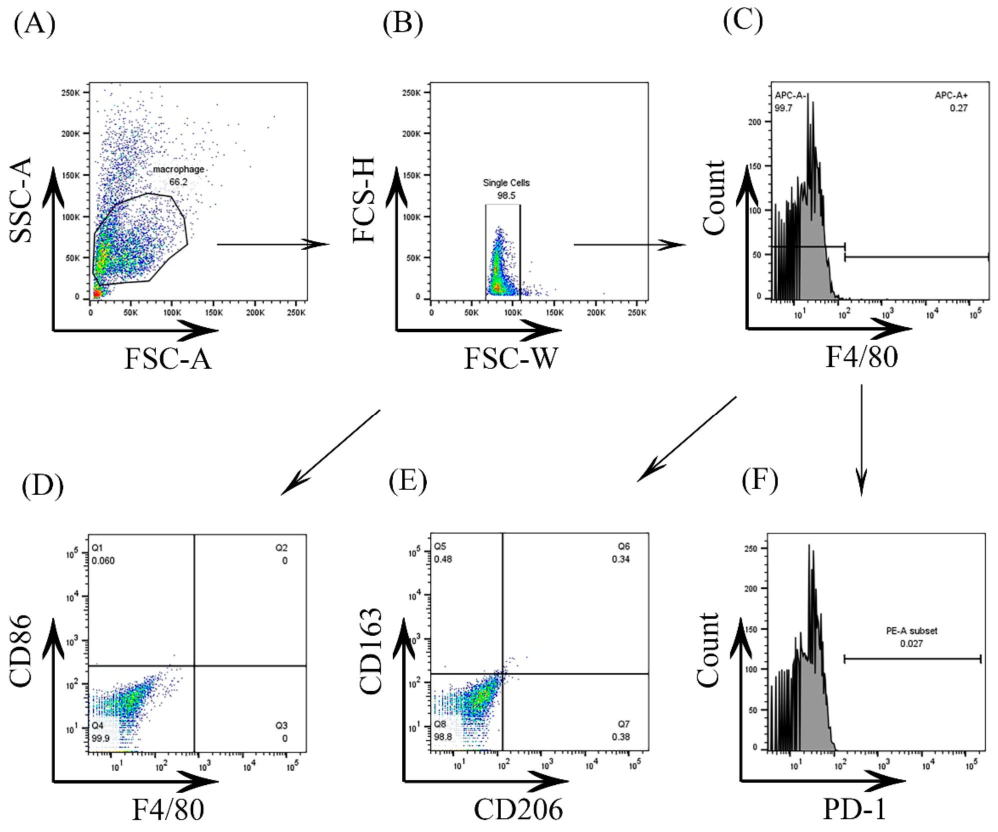

Fig.S2 Representative gating strategy for flow cytometric analysis of macrophage subsets and PD-1 expression.

A sequential gating strategy was applied to identify macrophage populations and assess their polarization states.

(A) Debris was excluded based on forward scatter area (FSC-A) and side scatter area (SSC-A). (B) Single cells were subsequently selected using an FSC-H versus FSC-A plot.

(C) Macrophages were identified by gating F4/80$^+$ cells. (D) Within the single-cell population, CD86 expression was analyzed in conjunction with F4/80 to define M1-like macrophages (F4/80$^+$CD86$^+$). (E) Within the F4/80$^+$ population, M2-like macrophages were identified based on co-expression of CD206 and CD163. (F) PD-1 expression was also assessed within the F4/80$^+$ gate to evaluate its expression on macrophages.

Supplementary Table S1

Gene	RefSeq Accession	Forward Primer (5'→3')	Reverse Primer (5'→3')	Forward Primer Position	Reverse Primer Position	Product Size (bp)
Gapdh	NM_001289726.2	CCTGGAGAAACCTGCCAAGTATG	TGTTGCTGTAGCCGTATTCATTGT	850-872	1073-1050	224
Il10	NM_010548.2	CTGAAGACCCTCAGGATGCG	ACACCTTGGTCTTGGAGCTTAT	413-432	528-507	116
Il1rn	NM_031167.5	CGTTGGAAGGCAGTGGAAGA	GGTTAGTATCCCAGATTCTGAAGGC	80-99	202-178	123
Tgfb	NM_011577.2	GACCGCAACAACGCCATCTAT	CGAAAGCCCTGTATTCCGTCTC	1210-1230	1520-1499	311
Vegfa	NM_009505.4	CTGCTGTAACGATGAAGCCCTG	GCTGTAGGAAGCTCATCTCTCC	1273-1294	1391-1370	119
Il1b	NM_008361.4	CCTTGTGCAAGTGTCTGAAGC	TCATCTTTTGGGGTCCGTCAAC	63-83	181-160	119
Il6	NM_031168.2	GAAATGAGAAAAGAGTTGTGCAATGG	ATATCCAGTTTGGTAGCATCCATCAT	268-293	390-365	123
Pdcd1	NM_008798.3	GCTCAACAAGTATGTCAGAGGC	AGCTCCTCATAGGCCACACTA	644-665	746-726	103
Alox15	NM_009660.3	AGTATCTCTGACCTCCCTGTAG	ATAACGGTGTCCATTGTCCC	495-516	589-570	95
Arg1	NM_007482.3	CATTGGCTTGCGAGACGTAGAC	GCTGAAGGTCTCTTCCATCACC	630-651	753-732	124
Ccl1	NM_011329.3	GCTTACGGTCTCCAATAGCTGC	GCTTTCTCTACCTTTGTTCAGCC	146-167	287-265	142

Table S1. PCR primer sequences
